# On the Practical Application of Chloroform as a Topical Anæsthetic to Mucous and Cutaneous Surfaces

**Published:** 1856-06

**Authors:** 

**Affiliations:** Edinburgh


### *On the Practical Application of Chloroform as a Topical Anæsthetic to Mucous and Cutaneous Surfaces.*

(From the unpublished works of Prof. Simpson, of Edinburgh.)

In 1848, my essay on local anæsthesia and its artificial production by chloroform, &c., was printed in two English medical journals.* In 1853, Dr. Hardy, of Dublin, published in the November number of the *Dublin Quarterly Journal of Medical Science*, an interesting communication on the same subject, entitled, "On the Local Application of the Vapor of Chloroform in the Treatment of various Diseases."

The principal peculiarity in Dr. Hardy's essay consisted in the proposal of a special valved instrument—the anæsthetic douche as he termed it†—for the purpose of applying in an intermittent stream, the vapor of chloroform to any part or surface that was wished to be affected.

But in projecting a stream of chloroform vapor upon any point, I have generally made use merely of a common enema syringe; and it will be found, I believe to serve as well, if not indeed better, than any of the complex and expensive special instruments invented for the purpose. In fact, a larger and more powerful stream of vapor can be kept up by an enema syringe than by any of the special anæsthetic douches which I have seen.

Any of the usual forms of pea-valve enema syringe will answer the purpose, provided their lower or receiving extremity be immersed in the vapor of chloroform, and the instrument worked in the usual way employed for the transmission of water or other liquids. The vapor of chloroform, &c., or rather of air loaded with the vapor, passes readily through the canal of the syringe, and is projected in an intermittent stream from its orifice.

The syringe which I have generally used for this purpose, is the barrel syringe of Mr. Higginson.‡ It consists of three pieces of caoutchouc tubing, the middle or thickest portion being provided at either extremity with the common pea or ball valve, and altogether forms, in my opinion, by far the simplest, most durable, and at the same time the cheapest description of syringe yet suggested for injecting fluids into the rectum or

* See Lancet and Medical Association Journal for July, 1848.

† Dr. Hardy's original Anæsthetic Douche "was formed of a caoutchouc bottle, having attached to one side of it a metallic chamber and egress pipe provided with two valves to regulate the admission and the egress of air and vapor. The metallic chamber was perforated at the side to admit a sponge sprinkled with chloroform, and this perforation was closed with a screw stopper." See figure of the instrument in Dublin Journal of Medical Science for November, 1853. Subsequently Dr. Hardy invented and used another Anæsthetic Douche, far more complex and expensive, of which he has given a description and drawing in the Dublin Medical Press for April, 1854. It was proposed to take out a patent for the douche.—(See Medical Pres for April 26, p. 268.)

‡ These syringes can be procured in this city, from Messrs. Codman & Co., Tremont street.—H. R. S.

vagina. When used for the transmission of chloroform vapor, it requires to be worked in the usual way for the transmission of liquids, but with its lower or inferior extremity placed in air loaded with the vapor of chloroform. In order to effect this last arrangement, all that is necessary is to place this lower extremity of the instrument in the neck of a phial or bottle containing liquid chloroform. The lower extremity of the barrel-enema syringe is generally made of the size and form of the two last joints of the little finger; and the tube is encircled with a projecting ridge or shoulder above this point. When employed as an anæsthetic douche, this finger-like end of the instrument is passed into the neck of a chloroform bottle sufficiently large to admit it easily; whilst at the same time the circular projecting ridge of the tube rests on the mouth of the phial. For this purpose the common six-ounce phial or bottle, with a mouth four or five lines wide, answers perfectly. An ounce of chloroform placed in the bottom of the phial will enable it to serve as an anæsthetic douche for a long time. Before using it, the shaking of the bottle will impregnate the air in it more thoroughly with chloroform vapor. When patients themselves employ the syringe and bottle, perhaps it will be found necessary to explain to them that they are not to inject the liquid chloroform through the tube, but only the vapor of it, or rather air loaded with the vapor.

The preceding simple arrangement converts a common enema or vaginal syringe into an anæsthetic douche, equally, or indeed more, powerful than the ingenious instrument specially invented by Dr. Hardy for the purpose. As a proof of this, let me merely state, that in various trials upon various individuals, I have never seen the stream of vapor from Dr. Hardy's instrument, when fully charged, produce a state of general anæsthesia when the jet from it was projected into the mouth; but I have found that result to follow in some instances when the same experiment was made with the stronger and more sustained stream of chloroform vapor sent through the common syringe.

When the inferior end of the enema syringe employed is of such a shape that it will not pass into the neck of a bottle containing chloroform, another arrangement may be required to supply it with chloroform vapor. For this purpose the lower end of the syringe may be placed upon the hollow of a concave sponge bedewed with chloroform; or a piece of lint, flannel, or the corner of a handkerchief, or other such material, freely wetted with it, may be lightly rolled around the lower extremity of the instrument. Sometimes, with the same view, I have placed the end of the syringe in the bottom of a cup or tumbler in which there was a bit of sponge or lint soaked with chloroform; for the vapor of chloroform being nearly four times heavier than atmospheric air, fills always the lower part of such a vessel. By any of these means a sufficient quantity of chloroform vapor can be supplied to fill the instrument, and to make a stream of it pass from its superior orifice, when the syringe is worked in the usual manner for transmitting liquids.

I have used the injection of chloroform vapor into the vagina by the preceding method, in many cases of painful and neuralgic conditions of the uterine and pelvic organs. In most instances, after the first sensations of warmth produced by the injection have passed away, relief has been found to follow for a greater or less length of time; and to sustain this state of freedom from suffering, the injection has generally required to be repeated by the patient after the lapse of a few hours. This treatment has appeared to me more particularly useful in neuralgic states of the uterine organs and passages; in those organic diseases that are occasionally accompanied with suffering, as carcinoma uteri; in some cases of severe feelings of bearing down, and incapacity to stand and walk, complicated with displacements and enlargements of the uterus; and in various spasmodic conditions of the uterus attended with pain, as in threatened abortions; in after-pains; and most markedly in severe dysmenorrhœa. But at the same time I would beg to remark that in various instances in which the preceding morbid states were present, and in which I fully expected the usual anodyne effect of the vapor to be experienced, the treatment has failed to give the usual relief; probably because the mere superficial anæsthesia which results from the anæsthetic vapor was not sufficient in depth or in degree to produce an anodyne effect. In other instances, on the contrary, in consequence, perhaps of the peripheral extremities of the nerves distributed to the genital mucous surface being specially affected, or having a special reflex influence upon the deeper-seated parts and pains, the chloroform vapor has succeeded in not only producing temporary relief, but in producing even a speedy and permanent cure, under circumstances where the previous duration and severity of the symptoms seemed, *a priori*, to forbid the hope of a restoration to health by this means alone. I had, for example, lately under my care, a patient who, in consequence of severe pelvic or uterine pain, had been obliged to keep the supine position upon the bed or sofa for nearly six months previously. All attempts at standing or walking brought on renewed paroxysms of suffering. The uterus was slightly retroverted, but otherwise appeared healthy. After being brought with some difficulty to Edinburgh from a distant part of England, the only treatment to which she was subjected consisted of the injection of chloroform vapor several times a day into the vagina, which at once relieved, and ultimately altogether removed, the uterine pains. Within a week, the morbid sensibility of the parts entirely disappeared. There was, about a month subsequently, a short relapse, in consequence of indiscretion in exercise and exposure to cold, but the attack speedily yielded to the same treatment. I never had the pleasure of watching such a speedy and perfect restoration to health and happiness from that state of hysteralgia which so often entails upon patients misery and suffering for long months and years.

I have repeatedly applied chloroform to the maternal passages during labor in cases of rigidity of these passages, and particularly in rigidity of the cervix uteri when coëxisting with morbid irritability and sensibility of the parts. In these instances I have used, sometimes a few drops of fluid chloroform, mixed up with oil, or with a small solid mass of butter or ointment. The practice has appeared to me to be followed by two beneficial results—first, the abatement of the supersensibility of the maternal canals; and secondly, very often also with an increased secretion of mucus and apparently an increased susceptibility to relaxation and dilatation in the rigid structures.*

* *Note on the mode of dilatation of the maternal passages in labor.*—During parturition the maternal canals, viz., the cervix uteri, vagina, and vulva, are no doubt dilated principally by the results of muscular uterine action and mechanical pressure. But these evidently become also dilatable and relaxed by another and an

In the preceding remarks I have hitherto spoken of chloroform when applied as a local anæsthetic to the genital mucous canals Its local anæsthetic action on other mucous surfaces has not yet been much studied. I have seen, however, the injection of the vapor of chloroform into the rectum, useful also in some instances of morbid irritability and sensibility in the lower end of the intestinal canal, in tenesmus, &c. The mucous membrane of the eye seems in most individuals—especially in its diseased states—too irritable to bear the contact of very concentrated chloroform vapor, such as I employed in some early experiments; but in cases of photophobia and supersensibility to light connected with scrofulous ophthalmia, &c., the vapor of cloroform, diluted with air or aqueous vapor, acts sometimes very markedly and beneficially as a local anæsthetic. I have seen the intolerance of light connected with ulcerative corneitis at once relieved by exposing the eye to the vapor of chloroform, raised by pouring a small quantity of the fluid into a cup of warm water. The patient will thus sometimes immediately be enabled to open the eye freely and without pain; the chloroform vapor often serves also as the best possible medicinal application to the ulcerated surface.† The dentist can occasionally relieve the pain of toothache, by the local anæsthesia resulting from the application of a drop of fluid chloroform to the exposed interior of the tooth; or by directing a stream of chloroform vapor upon it. In painful and spasmodic states of the respiratory canals, when chloroform is applied to their mucous surfaces by inhalation, it is difficult, or, indeed, impossible, to tell always whether the resulting relief is the effect of local or of general anæsthesia. In some cases of spasmodic asthma, relief is occasionally obtained by doses too slight to have acted by any general anæsthetic effects; but I have seen other instances of the same disease where the paroxysm was not effectually arrested till a complete state of general anæsthesia was produced. A similar observation holds true with regard to different cases of laryngismus. Sometimes that troublesome affection, hysterical or spasmodic aphonia, is at once cured

---

additional process, which is so far independent both of muscular action or mechanical pressure. In proof of this, we find the whole length of the canal of the vagina relaxing and widening during a protracted labor, *before* the head has yet passed the brim or fully opened the os uteri. This vital process of dilatation seems to me to consist of a rapid development of cells within the tissues of the walls of the maternal canals—just as the thick mucous secretion thrown out upon the free surface of these canals during labor (and indicative when present in great quantity, of great dilatability in these canals) is essentially, and in its ultimate physiological analysis, a rapid development of cells upon the free surface of their mucous coat. The application and stimulus of various substances, as simple warm water, of warm aqueous vapor, oils, simple or stimulant, &c., apparently promotes the dilatability of the tissues of the cervix uteri and vaginal canals, by promoting probably the more rapid formation of these cells. And from various cases which I have seen, I am led to believe that chloroform, both in the form of vapor, or of fluid diluted with oil or lard, will be found specially successful in producing this result, or at least—be the explanation what it may—in producing the required relaxation in cases of abnormal or morbid rigidity.

† The vapor of prussic acid carefully applied to the eye, by means of a proper cup or glass, acts probably upon the same principle. It was much recommended some years ago by Dr. Turnbull; but indiscriminately in almost all occular diseases. From what I have seen in practice, I believe that the use of dilute chloroform vapor and of carbonic acid will become common in affections of the cornea and conjunctiva connected with intolerance of light and supersensibility. They both act not as powerful local anæsthetics merely, but also as excellent medicinal applications to any existing ulcers, &c.

by a few inhalations of chloroform vapor, acting, perhaps, as much upon the principle of a local as of a general anæsthetic. The irritability of the cough in cases of phthisis, bronchitis, pneumonia, &c., is often effectually relieved by doses apparently too small to have acted otherwise than as local anæsthetics. Lastly, in reference to the topical anæsthetic influence of chloroform upon mucous membranes, let me add, that the swallowing of a few drops of liquid chloroform in oil, cream, soda water, or any other convenient vehicle, sometimes speedily abates nausea, vomiting, obstinate hiccough, &c.—perhaps upon the principle of its acting as a local and limited anæsthetic upon the walls of the stomach.

The preceding observations are limited to the local anæsthetic effect of chloroform upon mucous surfaces and canals. On the skin it produces a topical action, similar in principle, but far less in degree. When the epidermis is removed, or when the skin itself is destroyed, the surface of any existing sore, such as an irritable abrasion, an excoriated nipple, or a benign or carcinomatous ulcer, can be very remarkably anæsthetized and benumbed by the local application of chloroform vapor; but the feelings of great heat and pain, which in the first moments accompany its application, more than counterbalance, in most subjects the subsequent sedative affects derivable from its use. The various experiments which I have elsewhere detailed, show that chloroform fluid or vapor, when applied to the unbroken human skin, produces a degree and depth of local anæsthesia, that is sufficiently great to be sometimes useful in medicine, while it is not sufficiently great to be useful in operative surgery. In medicine, for example, the local anæsthetic effects of chloroform often prove most beneficial in local neuralgia, local rheumatism, &c.; and chloroform mixed with equal, or with varying parts of olive oil, according to the sensitiveness of the patient's skin is sometimes, in such cases, the most efficient form of cutaneous topical anodyne which we can employ. The amount of local anæsthesia, however, thus capable of being produced, is not, as I have just stated, by any means deep enough to enable the patient to endure any operative or surgical procedure. In the earlier part of 1854, however, a variety of experiments were made in the Parisian hospitals, under the full belief that a stream of chloroform vapor projected against the skin might produce such an amount of local anæsthesia, in any given part of the cutaneous surface, as would allow that surface to be cut or operated upon by the surgeon without pain to the patient. Dr. Hardy's anæsthetic douche, or some modification of it, was the instrument usually employed in these experiments. Several alleged cases of the perfect success of this local cutaneous anæsthesia were published in the French journals. It was averred, for example, that M. Dubois had opened with the knife, and without pain, an abscess in the axilla; that M. Nelaton opened an abscess in the foot—the vapor of chloroform having in each case been previously applied to the skin; and that M. Danyan, also without pain, made a caustic issue on the neck—the skin being prepared by the anæsthetic douche. But additional trials very speedily proved the inutility of the practice, as far at least as the possibility of producing by it immunity from the pain of surgical operations, was concerned. At the end of these trials, in commenting upon the subject in the Parisian hospitals, M. Latour, the learned editor of the *Union Medicale*, observes —"I have felt, I avow, distressed and humbled with all the noise that has been made, and with the recital of all the numerous experiments that have been tried in this matter. I have not desired to accumulate the record of them in this journal; and I wish that all trace of these facts were, for the honor of French physiology, blotted out as speedily as possible."*

In fact, the whole of these experiments and inquiries into the possibility of producing a sufficient amount of local anæsthesia for surgical purposes, by applying chloroform to the unbroken skin, resulted in the conclusion which I had already ventured to publish several years previously, namely, that "in the human subject, partial, and perhaps superficial local anæsthesia of a part, as the hand, can be produced by exposing it to the strong vapor of chloroform; but the resulting degree of local anæsthesia is *not* sufficiently deep to allow the part to be cut or operated on without pain."—*Boston Med. and Surg. Journ.*

* L'Union Medicale for 4th March, 1854.

